# Gutta-Percha

**Published:** 1897-10

**Authors:** 


### Abstracts and Translations.

### GUTTA-PERCHA.[1]

THE variation in the specific weight of gutta-percha is explained by Payen[2] as due to its porous structure, which varies according to its treatment.

Payen distended softened gutta-percha under strong pressure. The strips thus obtained he cut up under water into small pieces. Most of these little pieces sank at once; others floated for a time, until they had absorbed water, and then sank likewise. It was concluded from this that gutta-percha was apparently lighter than water only because of its numerous pores filled with air, and that this porosity diminished in proportion to the care with which the gutta-percha was purified.

---

[1] Extract translated from Handbuch der chemischen Technologie.

[2] Journal für praktische Chemie, lvii. 152.

For the purposes of purification the crude gutta-percha is dissolved in chloroform or bisulphide of carbon; the clouded liquid is first filtered under glass and then allowed to evaporate naturally in a shallow vessel. After the evaporation of the solvent the gutta-percha remains in the vessel as a thin skin, which can easily be removed if the vessel is plunged for a time into cold water and water is applied at the same time to its contents.

Exposed to air and light, gutta-percha rapidly undergoes a change, which is probably due to oxidation, at the same time giving off a sharp odor. This change in the gutta-percha is especially rapid if it is exposed to the air at a temperature of from 25° to 30° C., in the form of thin plates, strips, etc., if it is frequently moistened, and particularly if it is afterwards dried in the sunlight.

By this process it becomes brittle, crumbles like resin, gains in weight, is more readily soluble in alkali and alcohol, and even becomes a good conductor of electricity, which it is not in its normal state.

W. A. Müller[1] and A. W. Hofmann[2] regard this change as determined by oxidation. The oxidized portion of gutta-percha is insoluble in benzine, melts only at 100° C., and is supposed to be contained in commercial gutta-percha in proportions as large as fifteen per cent.

The composition of oxidized gutta-percha is, according to Müller,—

| | |
|---|---|
| Carbon | 76.15 |
| Hydrogen | 11.16 |
| Oxygen | 12.69 |

Gutta-percha which has become brittle by the action of light or air can be rendered serviceable again for many purposes by softening it in warm water and remoulding it; but it soon exhibits a tendency to crack.

Gutta-percha remains almost entirely unchanged if it is covered with water, especially sea-water, or if it is protected from the action of light. The readiness with which it suffers change in the air or under the action of light-rays materially limits its employment.

Gutta-percha resists the influence of most solvents. Concentrated solutions of alkalies, salt solutions, and dilute acids, even hydrofluoric acid, have no effect; absolute alcohol will dissolve by

---

[1] Chem. Soc. J., [2] iii. 273.

[2] Annal. d. Chem. u. Pharm., 215, 297.

boiling only a portion of those resinous bodies with their content of oxygen,—about fifteen or twenty per cent. According to Payen,[1] gutta-percha is only partially dissolved by ether. According to Arppe,[2] completely, especially if the ether is free from alcohol. Ether, to which some alcohol has been added, loses the property of completely dissolving gutta-percha. It is, however readily dissolved in bisulphide of carbon and chloroform, and, by the application of a mild warmth, also in benzine, the fugitive coal-tar oils, oil of turpentine, and the oils which are obtained from the dry distillation of rubber and gutta-percha.

The gutta-percha can be precipitated from a chloroform solution in the form of a white powder by the addition of ether. At low temperatures it is disintegrated by nitric acid, at the same time evolving a red vapor. Fuming sulphuric acid gradually imparts to it a brown tint; if longer exposed to the action of this acid, it swells and finally becomes a viscid mass. When warmed with sulphuric acid the gutta-percha at once becomes blackened, and suffers complete disintegration, carbon being separated from its other constituents. Strongly concentrated hydrochloric acid has but little influence on gutta-percha; only after being exposed to its action for a length of time does the latter become somewhat hard and brittle. The change which hydrochloric acid causes in gutta-percha has been observed in the gutta-percha tubes, which are used in drawing off the acid.

Gutta-percha is a poor conductor of heat and electricity: when rubbed against glass, flannel, etc., it becomes negatively electrified. Gutta-percha that has become grayish-blue by prolonged exposure to the air has, according to Riess,[3] the peculiar property of becoming positively electrified by friction. This superficial coating can, moreover, be removed by washing with ether or oil of turpentine; and if this is done upon one side of an old sheet of grayish-blue gutta-percha this side will be negatively electrified by friction with spun-glass or linen, while the other side when rubbed with the same substance will become positively electrified.

Under the polariscope gutta-percha exhibits beautiful color effects.

Pure gutta-percha is, like rubber, a hydrocarbon; indeed, both hydrocarbons are supposed to be isomeric. According to Sou-

---

1 Compt.-Rend., xxxv. 109; Jour. f. prakt. Chem., lxii. 243.

2 Dingl. pol. Journ., cxxi. 442.

3 Pogg. Ann., xci. 484; Dingl. pol. Journ., cxxvi. 115.

berain,[1] its formula is $C_6H_{10}$, corresponding to 87.8 of carbon and 12.2 of hydrogen. Souberain also found in crude gutta-percha a vegetable acid, extractive matter, and caseine, together with a resin, soluble in ether, and another resin, soluble in alcohol.

According to Müller,[2] the composition of commercial gutta-percha in one hundred parts is as follows:

| | |
|---|---|
| Pure gutta | 79.70 |
| Soft resin | 15.10 |
| Vegetable fibres | 2.18 |
| Moisture | 2.50 |
| Ashes | 0.52 |

From partially purified gutta-percha Arppe[3] secured six different resins, which showed a varying degree of solubility in ether and alcohol, according to the strength of the solvent and also according to the temperature. He arranged specific molecular formulæ for the different varieties of resin, but these are wholly hypothetical, as no compounds are known from which the atomic weights of these resins can be determined.

Payen[4] regards gutta-percha as consisting of three essentially different components. To these three substances he gives the names gutta, alban, and fluavil. They are supposed to occur in gutta-percha in the following average proportions:

| | |
|---|---|
| Gutta | 78 to 82 per cent. |
| Alban | 14 to 16 per cent. |
| Fluavil | 4 to 6 per cent. |

Of these three substances fluavil is soluble in cold alcohol; alban is soluble in boiling alcohol, and gutta-percha is insoluble. In order to separate them from each other the purified gutta-percha is treated for several hours with boiling alcohol and then filtered. In the course of a day or two numerous white opaline grains separate themselves from the alcoholic solution, especially at the sides and on the surface. These little spheres contain an inner kernel (of a yellow, amorphous mass) which may be dissolved in absolute spirits of wine. The outer shell, on the other hand, remains insoluble, and, after repeated treatment with the spirits of wine, becomes

[1] Journ. f. Pharm., 1847, 17; Dingl. pol. Journ., ciii. 415.
[2] Chem. Soc. J., iii. 273; Journ. f. prakt. Chem , xcvii. 380.
[3] Journ. f. prakt. Chem., liii. 171; Dingl. pol. Journ., cxxi. 442.
[4] Compt.-Rend., xxxv. 109; Dingl. pol. Journ., cxxvi. 115.

whiter and more transparent. By subjecting the granular mass to successive baths of cold alcohol, the fluavil is dissolved and the alban is left unaffected. When this has been separated by frequent boiling with alcohol, the final residuum is the gutta.

Fluavil is a yellowish, translucent resin, having a specific weight somewhat higher than 1. At 0° C. it is hard and inflexible; at 50° C. it becomes soft, at 60° C. it has the consistency of dough, and melts between 100° and 110° C. Under still greater heat it boils, and is finally decomposed, giving off hydrocarbons and acid fumes. It is soluble at low temperatures in ether, alcohol, oil of turpentine, bisulphide of carbon, and chloroform. When these solvents evaporate, it remains as an amorphous mass. It is not affected by dilute acids or concentrated alkalies, but is speedily decomposed by sulphuric or nitric acid.

According to Oudemans[1] its composition is—

| | |
|---|---|
| Carbon | 83.33 |
| Hydrogen | 11.11 |
| Oxygen | 5.55 |

corresponding to the formula $C_{20}H_{32}O$.

According to Oudemans, fluavil has been formed from the gutta by oxidation.

Alban forms a white, crystalline powder or even small grains. Under the microscope it appears as a series of transparent, radiating leaves. It begins to melt at 160° C., and becomes completely fluid without decomposition at 175° to 180° C.

In the presence of dilute acids and of alkalies its action is the same as that of fluavil; like the latter it is powerfully affected by concentrated sulphuric and nitric acids. In benzol, bisulphide of carbon, turpentine, chloroform, and absolute alcohol it is easily soluble; in ordinary spirits of wine it is soluble only at the boiling point.

Oudemans[2] considers alban as likewise produced by the oxidation of the pure gutta; he found its composition to be—

| | |
|---|---|
| Carbon | 78.9 |
| Hydrogen | 10.4 |
| Oxygen | 10.7 |

which corresponds to the formula $C_{20}H_{32}O_2$.

---

[1] Rep. d. Ch. Appl., 1858 u. 1859, 455; Jahresber. d. Chem., 1859, 517.

[2] Loc. cit.

Heated to 130° C. it is supposed to lose a molecule of water and thus to become $C_{20}H_{30}O$.

Gutta, the principal constituent of crude gutta-percha, becomes at 10° to 30° C. tough, flexible, and distensible, but not elastic; at 45° C. it becomes soft and assumes a darker tint. As the temperature increases it becomes more sticky and translucent. Between 100° and 110° C. it passes into a condition like dough; and, finally, at 130° C. it becomes a thin fluid, begins to boil, and throws off, as products of distillation, an oil and gaseous hydrocarbons.

In the presence of acids, spirits of wine, ether, and chloroform it acts as gutta-percha does. According to Arppe,[1] gutta is insoluble in ether only when it has been previously treated with alcohol.

When heated with nitric acid, it gives osmic acid and hydrocyanic acid in large quantities; when pulverized, it absorbs oxygen; hydrochloric acid gas changes it into a brownish black mass, which, owing to contraction, has the appearance of having been melted on the surface. It is extremely subject to change and is very difficult to keep without decomposition.

Its composition Oudemans[2] found to be—

| | |
|---|---|
| Carbon | 88 |
| Hydrogen | 11 |
| Oxygen | 0 |

corresponding to the formula $C_8H_5$ or $C_{20}H_{32}$.

E. H. von Baumhauer[3] has corroborated Oudemans's investigations concerning the elementary composition of gutta, alban, and fluavil. According to his statements, gutta-percha consists principally of a substance free from oxygen ($C_{20}H_{32}$, which is identical with the formula determined by Oudemans for gutta), and incidentally of several substances produced from the gutta by oxidation. Of these he believes that he has demonstrated two with certainty, one with the formula $C_{20}H_{32}O$ (the same as Oudemans's fluavil), and another having the formula $C_{20}H_{32}O_2$.

The strong tendency of gutta-percha to suffer change by the action of air and light and to become soft at 45° C. is partially counteracted by vulcanizing,—*i.e.*, by mixing it with sulphur.

---

[1] Journ. f. prakt. Chem., liii. 171; Dingl. pol. Journ., cxxi. 442.

[2] Rep. d. Ch. Appl., 1858 u. 1859, 455; Jahresber. d. Chem., 1859, 517.

[3] Journ. f. pr. Chem., lxxviii. 277.

